# Supplementary material for: Pangolin hunting in southeast Nigeria is motivated more by local meat consumption than international demand for scales
Source: Nat Ecol Evol. 2025 Jun 13;9(8):1349–58. doi: 10.1038/s41559-025-02734-3 (PMC12328206; doi:10.1038/s41559-025-02734-3)
Supplement: Supplementary file 1 — Supplementary text, Figs. 1–4, Tables 1–4 and extended research credits. [file 41559_2025_2734_MOESM1_ESM.pdf]

nature ecology & evolution

Supplementary information

https://doi.org/10.1038/s41559-025-02734-3

Pangolin hunting in southeast Nigeria is motivated more by local meat consumption than international demand for scales

In the format provided by the authors and unedited

The file includes:

- Supplementary Text
- Supplementary Figure 1-4
- Supplementary Tables 1-4
- Extended Research Credits

Supplementary Text

Comparison of Species Extraction Rates

Our comparison of species extraction rates involved five species: black-bellied pangolin (*Phataginus tetradactyla*), white-bellied pangolin (*P. tricuspis*), African brush-tailed porcupine (*Atherurus africana*), blue duiker (*Philantomba monticola*), red river hog (*Potamochoerus porcus*). We compared extraction rates per season reported in the present study with the number of individuals reportedly captured each season in a separate protocol in which we tracked formal hunters continuously over three years in the same landscape [1].

The latter data (n = 1,445 records across species) were gathered from 33 male hunters across two communities in the Cross River Forest landscape from April 2020 to March 2023. We recruited hunters through community hunter associations, specifically targeting formal hunters (those primarily using guns), as the activities of those who predominantly use snares are more diffuse and hence harder to follow. We then deployed questionnaires through trained local field assistants to collect data from hunters after every hunting trip. The data collected included the number and species of animals captured, which we compared with the estimates provided by hunters in the current study using the median values per season (fig. S7). The complete protocol we used to collect the hunter follows data is detailed in Emogor et al. 2025 [1].

Mass of Pangolin Derivatives

We estimated the median mass of dried pangolin meat and scales from already-existing data collected from our study landscape. For meat, we used 24 records of whole, dried, and descaled carcasses of black- and white-bellied pangolins (n = 2 and n = 22, respectively). We obtained these data during market surveys in two wild meat markets in southeast Nigeria from August 2020 to November 2023. For scales, we used 10 records from whole pangolins, which we derived from the hunter-monitoring work described above (n = 4 black-bellied and n = 6 white-bellied). Upon indicating our interest in obtaining the mass of pangolin scales to the hunters, local research assistants periodically received donations of pangolin carcasses, which were descaled, with all the scales on a carcass dried and weighed. We returned meat and scales to the hunters in all cases. The median mass per part is shown in Table S6.

Specifications of Price Models

The equation for the model predicting the variation in prices of pangolin parts is given by

$$log(Real\ price_{ijkl}) = \beta_0 + \beta_1 Part_i * \beta_2 Period_j + \beta_3 Respondent\ type_k + \beta_4 Species_l + \alpha_{ij} \quad (1)$$

where $Real\ prices_{ijkl}$ is the inflation-adjusted price of part *i* in period *j*, with the prices provided by respondent *k* for species *k*; β_0 is the intercept; β_{1-5} are the slopes of the respective predictors; and α_{ij} is a random intercept (specified in R as $(1|Location) + (1|household.id/respondent.id)$). We assume that the errors follow a Gaussian distribution.

The equation for the model predicting the variation in prices of African brush-tailed porcupine, blue duiker and red river hog meat is given by

$$log(Real\ price_{ij}) = \beta_0 + \beta_1 Species_j + \beta_1 Period_j + \beta_2 Respondent\ type_k + \alpha_{ij} \quad (2)$$

where $Real\ prices_{ij}$ is the inflation-adjusted price of species *i* in period *j* provided by respondent *k* ; β_0 is the intercept; β_{1-5} are the slopes of the respective predictors; and α_{ij} is random intercept (specified in R as $(1|Location) + (1|household.id/respondent.id)$). Again we assume that the errors follow a Gaussian distribution.

References

1. Emogor, C. A. *et al.* Predictors of Frequency and Success of Wild Meat Hunting Trips and Carcass Prices in an African Biodiversity Hotspot. *Hum Ecol* (2025) doi:10.1007/s10745-025-00572-2.

Participant Information Sheet

Department of Zoology
Address: Downing Street, Cambridge, CB2 3EJ, United Kingdom
Email: reception@zoo.cam.ac.uk
Tel: +44 (0)1223 336600

Understanding the drivers and dynamics of pangolin exploitation

Before you decide to take part in this study it is important for you to understand why the research is being done and what it will involve. Please take time to read the following information carefully and discuss it with others if you wish. A member of the team can be contacted if there is anything that is not clear or if you would like more information. Take time to decide whether or not you wish to take part.

Purpose of the study

Pangolins are unique animals. They are hunted alongside other wild animals for food and income, but we know little about why, how, and other drivers of their exploitation. This work will allow us gather useful information to understand how drivers of pangolin exploitation has changed over space and time.

Why have I been chosen?

You have been selected to partake in this study because you either hunt wild animals for food or income or you sell meat and other derivatives from wild animals.

Do I have to take part?

Your participation is entirely voluntary, and if you decide to take part, and you are free to stop the interview at any time. However, please note that, given your responses are not linked to you, we are unable to exclude your responses at the end of the interview should you decide to drop from the study at that time.

What will happen to me if I take part?

We will ask you a series of questions for about 30 minutes. The interview will not be recorded. We will use a standardised form installed in a mobile tablet.

Are there possible disadvantages and/or risks in taking part?

Data collection sessions can be time consuming and will retire you to think hard about the last five years. Additionally, the information you will provide might threaten your livelihood as our published results could be used by relevant authorities to curb pangolin exploitation.

What are the possible benefits of taking part?

Your participation will provide useful information needed to conserve wild animals and protect your livelihoods and the livelihoods of other local people that depend on wild animals for food and income. Lastly, you will receive a thank-you souvenir each month until the end of the study.

Will my taking part in this project be kept confidential?

All data will be identified only by a code, with personal details kept in a locked file or secure computer with access only by the immediate research team. We understand you might be concerned about the sensitivity of the information you will be providing especially the implications of this data getting to law enforcement agents. Please be assured that we will not share the information you provide (in part or whole) with a third party. Please follow the link below for general information about how the University of Cambridge uses personal data: https://www.information-compliance.admin.cam.ac.uk/data-protection/research-participant-data.

What will happen to the results of the research project?

Results will be presented at conferences and written up in journals. Results are normally presented in terms of groups of individuals. If any individual data are presented, the data will be anonymous, without any means of identifying the individuals involved. We will likely use this data for subsequent research.

Who is organising and funding the research?

This study is funded by the Gates Cambridge Trust (University of Cambridge).

Ethical review of the study

This project has been reviewed by the University of Cambridge Psychology Research Ethics Committee. Additionally, a comprehensive risk assessment has been carried out for the researchers including assistants and participants who volunteer to take part in this study.

Contact for further information

If you decide to take part, please contact Charles Emogor (cae37@cam.ac.uk or 081*). Thank you.

*Redacted phone number.

Consent Form

UNIVERSITY OF CAMBRIDGE

Department of Zoology
Address: Downing Street, Cambridge, CB2 3EJ, United Kingdom
Contact: reception@zoo.cam.ac.uk | +44 (0)1223 336600

Consent Form: Understanding The Drivers and Dynamics of Pangolin Hunting and Use

Please tick the boxes below as appropriate.

- ☐ *I confirm that I have read and understood the Participant Information Sheet*
- ☐ *I have had the opportunity to ask questions and had them answered*
- ☐ *I understand that all personal information will remain confidential and that all efforts will be made to ensure I cannot be identified (except as might be required by law)*
- ☐ *I understand that the data will be stored anonymously and securely, and may be used for future research*
- ☐ *I understand that my participation is voluntary and that I am free to withdraw at any time without giving a reason*
- ☐ *I agree to take part in this study*

Location (including community name): ______________________________________

Participant's signature: ______________________ Date: ____________________

Investigator's name: ___________________________________ Signature: ________________

Date: _____________________

Supplementary Figures

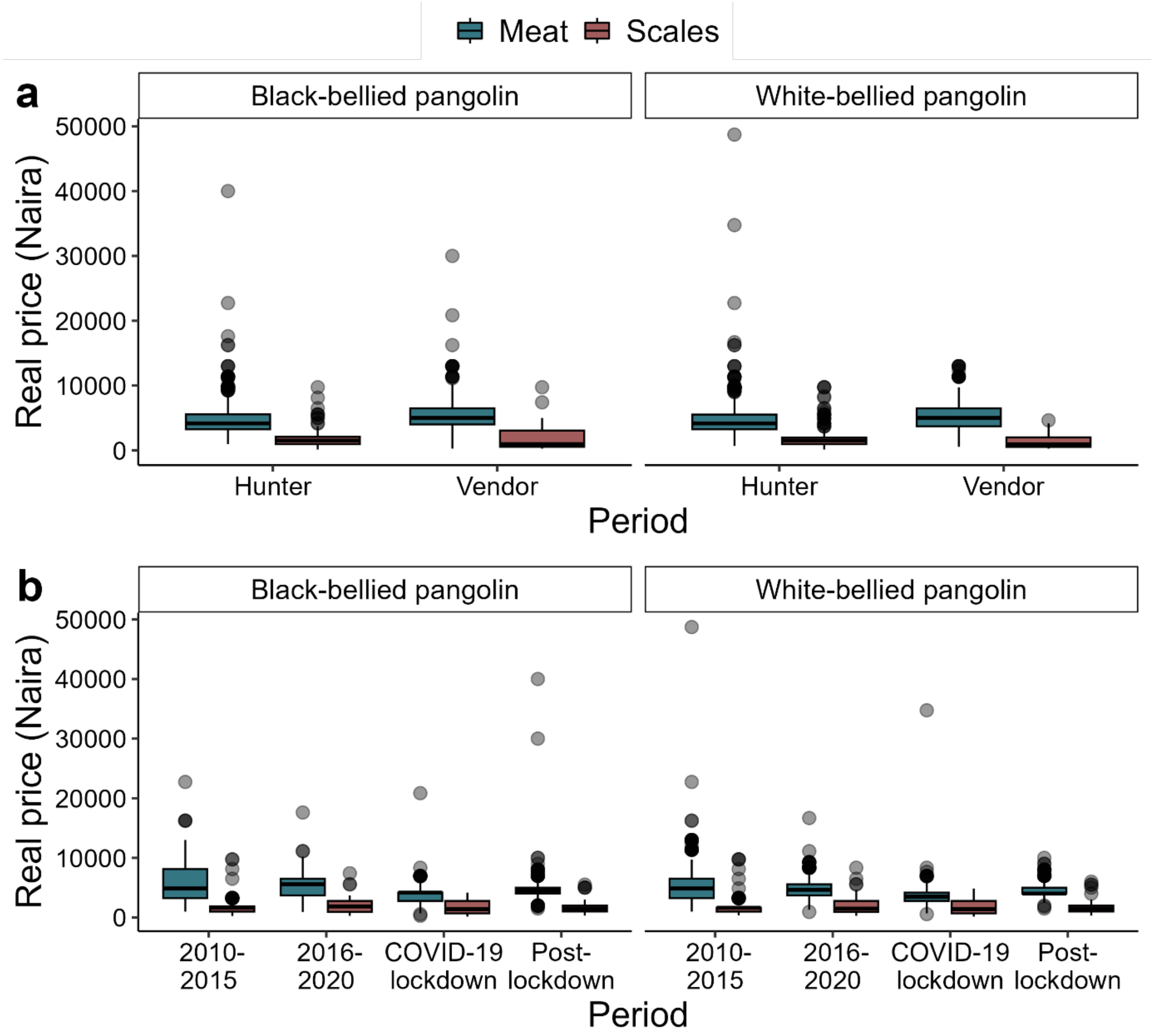

Supplementary Figure 1 | The distribution of real prices of whole meat and scales from black- and white-bellied pangolins provided by hunters and vendors (a) and the prices for each part per period (b). The thick horizontal bar in each box shows the median, the box the interquartile range, and the lines the overall range, with outliers marked by black circles (with darker colours signifying relatively higher counts).

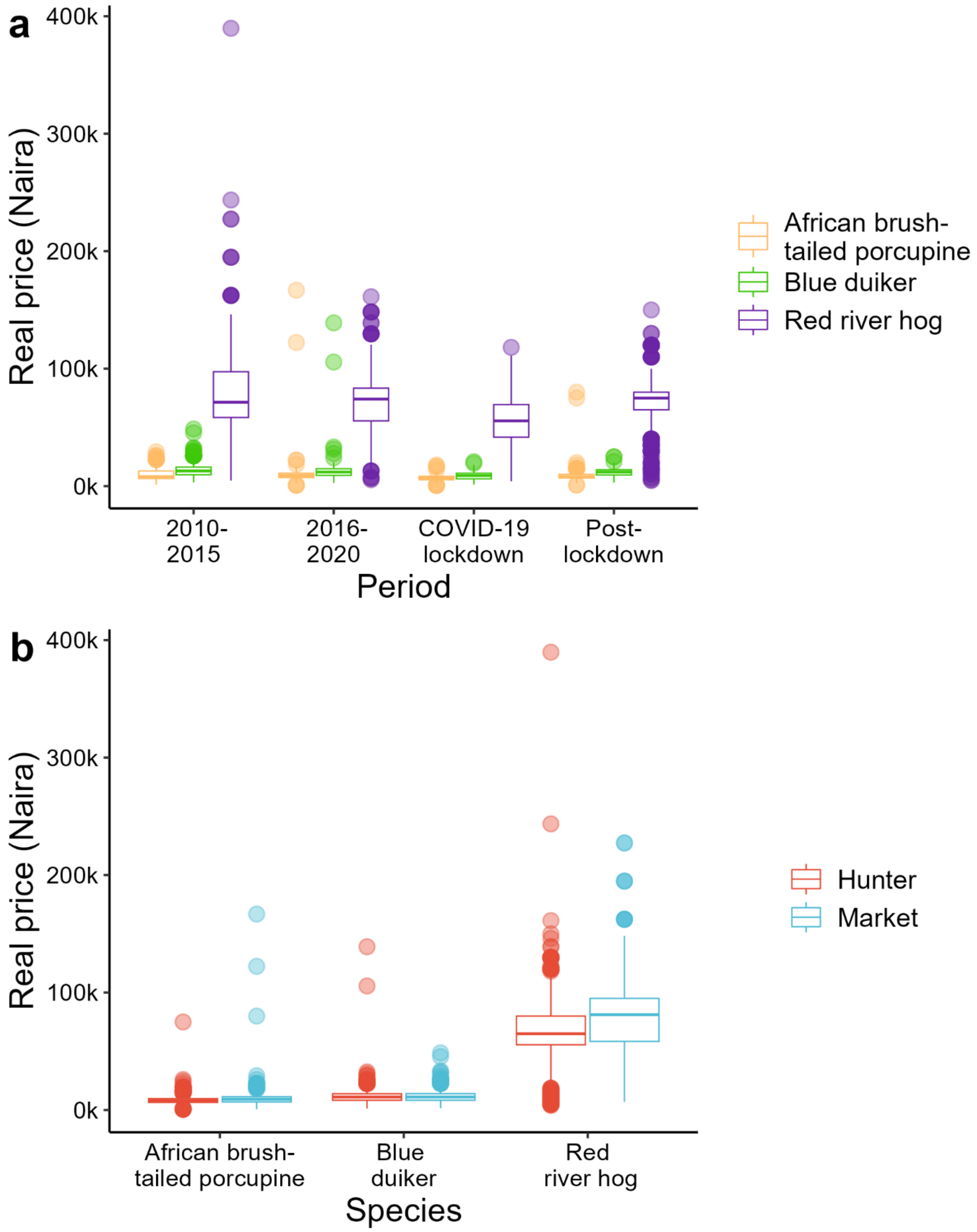

Supplementary Figure 2 | The real price over time of whole carcasses of African brush-tailed porcupine, blue duiker, and red river hog combined for hunters and vendors (a) and prices per species shown separately for vendors and hunters (b). The thick horizontal bar in each box shows the median, the box the interquartile range, and the lines the overall range, with outliers marked by circles (with darker colours signifying relatively higher counts).

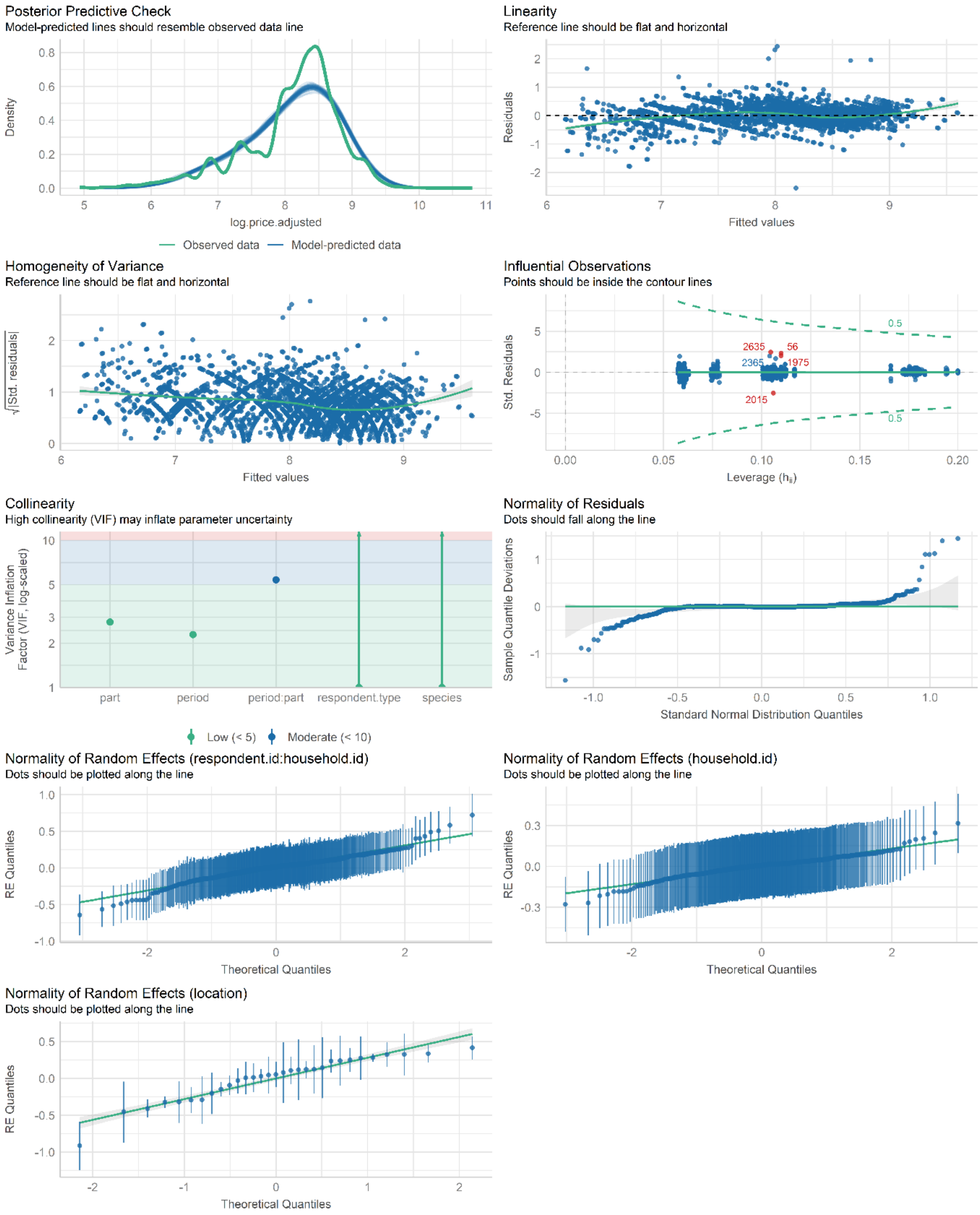

Supplementary Figure 3 | Diagnostics of the model predicting the real prices of pangolin parts over time. Diagnostic parameters and interpretation of the plot are provided on top of each panel. Model assessment was conducted using Performance package in R.

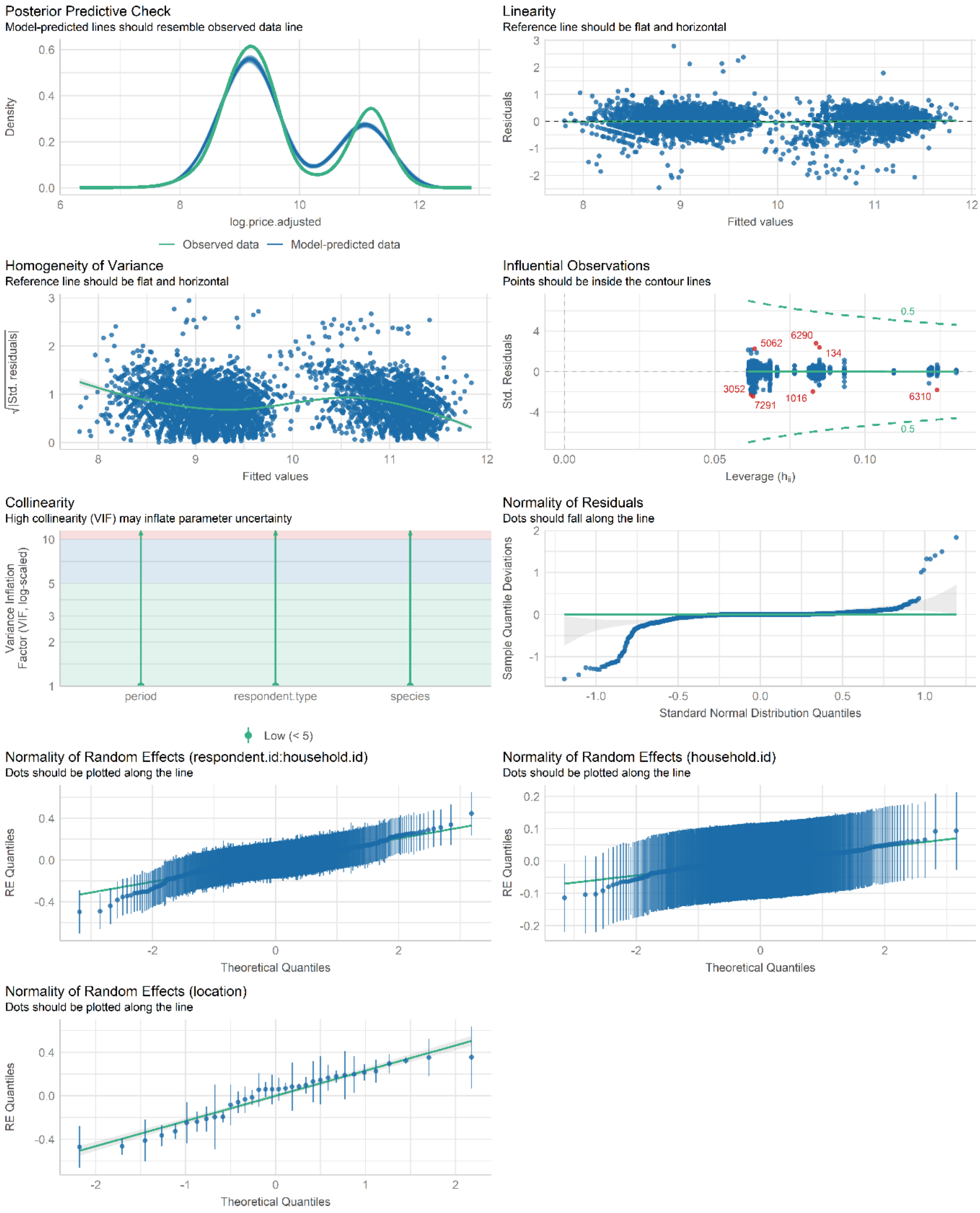

Supplementary Figure 4 | Diagnostics of the model predicting trends in the real prices of African brush-tailed porcupine, blue duiker, and red river hog. Diagnostic parameters and interpretation of the plot are provided on top of each panel. Model assessment was conducted using Performance package in R.

Supplementary Tables

Supplementary Table 1a: Gaussian-based mixed effects model of the price of whole meat and scales of adult black- and white-bellied pangolins. Number of observations: 3,960; Respondent ID: Household ID: 431, Household ID: 392; Location: 31.

Random effect:

Groups	Term	Variance	Std. dev
Respondent ID: Household ID	Intercept	0.046	0.214
Household ID	Intercept	0.019	0.138
Location	Intercept	0.105	0.325
Residual		0.107	0.328

Fixed effects:

Term	Estimate (*β*)	Standard error (*SE*)	Degree of freedom (DF)	T ratio	P value
Intercept	8.481	0.069	32.798	123.630	< .0001
Period: Jan 2016-Feb 2020	-0.112	0.017	3529.252	-6.635	< .0001
Period: April-Sept 2020	-0.449	0.017	3529.252	-26.566	< .0001
Period: Oct 2020-Sept 2023	-0.179	0.017	3529.252	-10.568	< .0001
Part: Scales	-1.176	0.026	3680.101	-44.657	< .0001
Species: White-bellied	0.010	0.011	3609.105	0.947	0.344
Respondent type: Vendor	0.053	0.046	430.718	1.167	0.244
Jan 2016-Feb 2020 (Period): Scale (Part)	0.109	0.034	3529.252	3.173	0.002
April-Sept 2020 (Period): Scale (Part)	0.255	0.034	3529.252	7.405	< .0001
Oct 2020-Sept 2023 (Period): Scale (Part)	0.116	0.034	3529.252	3.379	< .0001

Supplementary Table 1b: Pairwise post-hoc (Tukey test) comparing the average price of whole meat and scales from adult black- and white-bellied pangolins in each period. We accounted for multiple comparison using the Benjamini-Hochberg procedure, which controls false discovery rate.

Pair	*β*	*SE*	DF	T ratio	P value
Jan 2010-Dec 2015					
Meat–Scales	1.176	0.026	3676	44.644	<.0001
Jan 2016-Feb 2020					
Meat–Scales	1.067	0.026	3676	40.500	<.0001

April-Sept 2020					
Meat—Scales	0.921	0.026	3676	34.973	<.0001
Oct 2020-Sept 2023					
Meat—Scales	1.060	0.026	3676	40.230	<.0001

Supplementary Table 1c: Pairwise post-hoc (Tukey test) comparing the average price between periods for whole meat and scales from adult black- and white-bellied pangolins. We accounted for multiple comparison.

Pair	*β*	*SE*	DF	T ratio	P value
Meat					
Jan 2010-Dec 2015—Jan 2016-Feb 2020	0.112	0.017	3523	6.635	< .0001
Jan 2010-Dec 2015—April-Sept 2020	0.449	0.017	3523	26.566	< .0001
Jan 2010-Dec 2015—Oct 2020-Sept 2023	0.179	0.017	3523	10.568	< .0001
Jan 2016-Feb 2020—April-Sept 2020	0.337	0.017	3523	19.931	< .0001
Jan 2016-Feb 2020—Oct 2020-Sept 2023	0.066	0.017	3523	3.933	0.0001
April-Sept 2020—Oct 2020-Sept 2023	-0.270	0.017	3523	-15.997	< .0001
Scales					
Jan 2010-Dec 2015—Jan 2016-Feb 2020	0.003	0.030	3523	0.100	0.9204
Jan 2010-Dec 2015—April-Sept 2020	0.194	0.030	3523	6.485	< .0001
Jan 2010-Dec 2015—Oct 2020-Sept 2023	0.062	0.030	3523	2.082	0.0561
Jan 2016-Feb 2020—April-Sept 2020	0.191	0.030	3523	6.385	< .0001
Jan 2016-Feb 2020—Oct 2020-Sept 2023	0.059	0.030	3523	1.982	0.0571
April-Sept 2020—Oct 2020-Sept 2023	-0.132	0.030	3523	-4.403	< .0001

Supplementary Table 2a: Gaussian-based mixed effects model of the price of carcass of African brush-tailed porcupine, blue duiker, and red river hog. Number of observations: 7,916; Respondent ID: Household ID: 690, Household ID: 621; Location: 34.

Random effect:

Group	Term	Variance	Std. dev
Respondent ID: Household ID	Intercept	0.020	0.141
Household ID	Intercept	0.004	0.064
Location	Intercept	0.062	0.249
Residual		0.103	0.322

Fixed effects:

Term	β	*SE*	DF	T ratio	P value
Intercept	9.025	0.047	36.469	193.152	< 0.001
Period: Jan 2016-Feb 2020	-0.063	0.010	7212.499	-6.190	< 0.001
Period: April-Sept 2020	-0.368	0.010	7212.499	-35.958	< 0.001
Period: Oct 2020-Sept 2023	-0.070	0.010	7212.499	-6.876	< 0.001
Species: Blue duiker	0.271	0.009	7244.707	30.971	< 0.001
Species: Red river hog	2.064	0.009	7340.642	229.060	< 0.001
Respondent type: Vendor	0.127	0.021	665.618	5.956	< 0.001

Supplementary Table 2b: Pairwise post-hoc Tukey test showing temporal changes in the price of the carcass of African brush-tailed porcupine, blue duiker, and red river hog. We accounted for multiple comparison using the Benjamini-Hochberg procedure, which controls false discovery rate.

Pair	β	*SE*	DF	T ratio	P value
Jan 2010-Dec 2015—Jan 2016-Feb 2020	0.063	0.010	7223	6.190	< 0.001
Jan 2010-Dec 2015—April-Sept 2020	0.368	0.010	7223	35.958	< 0.001
Jan 2010-Dec 2015—Oct 2020-Sept 2023	0.070	0.010	7223	6.876	< 0.001
Jan 2016-Feb 2020—April-Sept 2020	0.304	0.010	7223	29.767	< 0.001
Jan 2016-Feb 2020—Oct 2020-Sept 2023	0.007	0.010	7223	0.685	0.493
April-Sept 2020—Oct 2020-Sept 2023	-0.297	0.010	7223	-29.082	< 0.001

Supplementary Table 2c: Pairwise post-hoc Tukey test comparing the price trend for carcasses of African brush-tailed porcupine, blue duiker, and red river hog. We accounted for multiple comparison.

Pair	β	*SE*	DF	T ratio	P value
Porcupine—Blue duiker	-0.271	0.009	7255	-30.971	< 0.001
Porcupine—Red river hog	-2.064	0.009	7350	-229.047	< 0.001
Blue duiker—Red river hog	-1.793	0.009	7350	-199.161	< 0.001

Supplementary Table 3a: Household census questionnaire used during the hunter and vendor behaviour survey. The questionnaire we deployed via KoboToolbox (https://www.kobotoolbox.org/) installed in mobile tablets.

Questions	Options/text field	Note
What is the name of the community?		We have anonymised this list in line with our ethics requirement.
What is the household ID?		Use the following to set the household ID: Community name + unique number (without the plus sign and without spaces). For example, if interviewing the third household in Calabar, the ID will be CAL03. The tenth house in Abuja will be ABU010.<br><br>Write the household ID on a sticker, attach it to the house, and then move on to the next.
What is the total number of people in household?		
How many gun hunters live in the household?		Ask to see the gun or evidence of gun ownership if possible.
How many trappers live in the household?		
How many wild meat traders live in the household?		

Supplementary Table 3b: The primary questionnaire used during the hunter and vendor behaviour survey. The questionnaire we deployed via KoboToolbox (https://www.kobotoolbox.org/) installed in mobile tablets.

Questions	Options/text field	Note
What is the name of the community or market?		We have anonymised this list in line with our ethics requirement.
What category of respondent are you interviewing?	1. Hunter<br>2. Vendor	
What category of hunter are you interviewing?	1. Formal (or gun hunter)<br>2. Casual	
What is the household ID?		This ID should match the ID in the household census questionnaire for this household.
What is the respondent ID?		Use the following to set the respondent ID: Surveyor code + community code + unique number (without the plus sign and without spaces). For example, if Charles was interviewing the third person in Calabar, the ID will be CHACAL03. The third interview in Abuja conducted by Andrew will be: ANDABU10.
What is the respondent's sex?	1. Female<br>2. Male	
What is the year of birth of the respondent?		You can only interview people above 18 years. The form rejects any entry below 2006.
Prices (hunter only)		Stress that you mean the price that they or their colleagues sold meat and scales for. Use "999" if respondent

			does not know the price for each period.
What was the average/regular/usual price of the whole meat from an adult African brush-tailed porcupine in the following periods…	1. January 2010-December 2015		
	2. January 2016-March 2020		2010-15 was when Goodluck Jonathan was president.
	3. COVID lockdown (April-September 2020)		2016-19 was the majority of Buhari's first term in office.
	4. After lockdown (October 2020 to date)		
What was the average/regular/usual price of the whole meat from an adult blue duiker in the following periods…	1. January 2010-December 2015		
	2. January 2016-March 2020		2010-15 was when Goodluck Jonathan was president.
	3. COVID lockdown (April-September 2020)		2016-19 was the majority of Buhari's first term in office.
	4. After lockdown (October 2020 to date)		
What was the average/regular/usual price of the whole meat from an adult red river hog in the following periods…	1. January 2010-December 2015		
	2. January 2016-March 2020		2010-15 was when Goodluck Jonathan was president.
	3. COVID lockdown (April-September 2020)		2016-19 was the majority of Buhari's first term in office.

	4. After lockdown (October 2020 to date)		
What was the average/regular/usual price of the whole meat from an adult black-bellied pangolin in the following periods…	1. January 2010-December 2015		
	2. January 2016-March 2020		2010-15 was when Goodluck Jonathan was president.
	3. COVID lockdown (April-September 2020)		2016-19 was the majority of Buhari's first term in office.
	4. After lockdown (October 2020 to date)		
What was the average/regular/usual price of the whole scales from an adult black-bellied pangolin in the following periods…	1. January 2010-December 2015		
	2. January 2016-March 2020		2010-15 was when Goodluck Jonathan was president.
	3. COVID lockdown (April-September 2020)		2016-19 was the majority of Buhari's first term in office.
	4. After lockdown (October 2020 to date)		
What was the average/regular/usual price of the whole meat from an adult white-bellied pangolin in the following periods…	1. January 2010-December 2015		
	2. January 2016-March 2020		2010-15 was when Goodluck Jonathan was president.
	3. COVID lockdown (April-September 2020)		2016-19 was the majority of Buhari's first term in office.

	4. After lockdown (October 2020 to date)		
What was the average/regular/usual price of the whole scales from an adult white-bellied pangolin in the following periods…	1. January 2010-December 2015		
	2. January 2016-March 2020		2010-15 was when Goodluck Jonathan was president.
	3. COVID lockdown (April-September 2020)		2016-19 was the majority of Buhari's first term in office.
	4. After lockdown (October 2020 to date)		
Uses of pangolin meat (hunter and vendor)			Use 10 beans to get proportions for each period.
What did you do with meat from the pangolin you caught in the following periods…	1. January 2010-December 2015	1. Eat at home<br>2. Sell<br>3. Medicine at home (including voodoo or *juju*)	
	2. January 2016-March 2020	1. Eat at home<br>2. Sell<br>3. Medicine at home (including voodoo or *juju*)	2010-15 was when Goodluck Jonathan was president.
	3. COVID lockdown (April-September 2020)	1. Eat at home<br>2. Sell<br>3. Medicine at home (including voodoo or *juju*)	2016-19 was the majority of Buhari's first term in office.

	4. After lockdown (October 2020 to date)	1. Eat at home<br>2. Sell<br>3. Medicine at home (including voodoo or *juju*)	
	Uses of pangolin scales (hunter only)		Use 10 beans to get proportions for each period.
What did you do with the scales from the pangolin you caught in the following periods…	1. January 2010-December 2015	1. Sell<br>2. Medicine at home<br>3. Discard (throw away or burn)	
	2. January 2016-March 2020	1. Sell<br>2. Medicine at home<br>3. Discard (throw away or burn)	2010-15 was when Goodluck Jonathan was president.
	3. COVID lockdown (April-September 2020)	1. Sell<br>2. Medicine at home<br>3. Discard (throw away or burn)	2016-19 was the majority of Buhari's first term in office.
	4. After lockdown (October 2020 to date)	1. Sell<br>2. Medicine at home<br>3. Discard (throw away or burn)	
	Uses of pangolin scales (vendor only)		Use 10 beans to get proportions for each period.
What did you do with the scales from the pangolin you caught in the following periods…	1. January 2010-December 2015	1. Sell<br>2. Medicine at home<br>3. Discard (throw away or burn)<br>4. No access to scales	
	2. January 2016-March 2020	1. Sell<br>2. Medicine at home<br>3. Discard (throw away or burn)<br>4. No access to scales	2010-15 was when Goodluck Jonathan was president.

	3. COVID lockdown (April-September 2020)	1. Sell<br>2. Medicine at home<br>3. Discard (throw away or burn)<br>4. No access to scales	2016-19 was the majority of Buhari's first term in office.
	4. After lockdown (October 2020 to date)	1. Sell<br>2. Medicine at home<br>3. Discard (throw away or burn)<br>4. No access to scales	
	Reasons for hunting pangolins (hunter only)		Use 10 beans to get proportions for each period.
What was the main reason for hunting pangolins in the following periods…	1. January 2010-December 2015	1. Food for household (meat)<br>2. Sell meat for money<br>3. Sell scales for money<br>4. Use scales for medicine	
	2. January 2016-March 2020	1. Food for household (meat)<br>2. Sell meat for money<br>3. Sell scales for money<br>4. Use scales for medicine	2010-15 was when Goodluck Jonathan was president.
	3. COVID lockdown (April-September 2020)	1. Food for household (meat)<br>2. Sell meat for money<br>3. Sell scales for money<br>4. Use scales for medicine	2016-19 was the majority of Buhari's first term in office.
	4. After lockdown (October 2020 to date)	1. Food for household (meat)<br>2. Sell meat for money<br>3. Sell scales for money<br>4. Use scales for medicine	
	Motivations for hunting pangolins (hunter only)		Use 10 beans to get proportions for each period.
How did you catch pangolins in the following periods…	1. January 2010-December 2015	1. Hunting for wild animals (bushmeat hunting)	

		2. Opportunistic (when not hunting. E.g. while farming)<br>3. Specifically hunting for pangolins (Pangolin hunting)<br>4. Paid by someone to catch pangolins	
	2. January 2016-March 2020	1. Hunting for wild animals (bushmeat hunting)<br>2. Opportunistic (when not hunting. E.g. while farming)<br>3. Specifically hunting for pangolins (Pangolin hunting)<br>4. Paid by someone to catch pangolins	2010-15 was when Goodluck Jonathan was president.
	3. COVID lockdown (April-September 2020)	1. Hunting for wild animals (bushmeat hunting)<br>2. Opportunistic (when not hunting. E.g. while farming)<br>3. Specifically hunting for pangolins (Pangolin hunting)<br>4. Paid by someone to catch pangolins	2016-19 was the majority of Buhari's first term in office.
	4. After lockdown (October 2020 to date)	1. Hunting for wild animals (bushmeat hunting)<br>2. Opportunistic (when not hunting. E.g. while farming)<br>3. Specifically hunting for pangolins (Pangolin hunting)<br>4. Paid by someone to catch pangolins	
	Hunting methods (hunter only)		Use 10 beans to get proportions for each period.
What methods did you use to hunt pangolins in the following periods…	1. January 2010-December 2015	1. Gun<br>2. Trapping (e.g. wire snare)<br>3. Pickup	

		4. Dog (including pickup or gun)	
	2. January 2016-March 2020	1. Gun<br>2. Trapping (e.g. wire snare)<br>3. Pickup<br>4. Dog (including pickup or gun)	2010-15 was when Goodluck Jonathan was president.
	3. COVID lockdown (April-September 2020)	1. Gun<br>2. Trapping (e.g. wire snare)<br>3. Pickup<br>4. Dog (including pickup or gun)	2016-19 was the majority of Buhari's first term in office.
	4. After lockdown (October 2020 to date)	1. Gun<br>2. Trapping (e.g. wire snare)<br>3. Pickup<br>4. Dog (including pickup or gun)	
	Number of pangolins caught (hunter only)		If a respondent did not catch any pangolins in the last two years, please just add "999" to submit the form.
Roughly how many of the following animals did you catch in the dry season (November-March) in last two years?	1. Black-bellied pangolin		
	1. White-bellied pangolin		
	2. African brush-tailed porcupine		
	3. Red river hog		
	4. Blue duiker		
	Number of pangolins caught (hunter only)		If a respondent did not catch any pangolins in the last two years, please just add "999" to submit the form.

Roughly how many of the following animals did you catch in the wet season (April-December) in last two years?	2. Black-bellied pangolin	
	5. White-bellied pangolin	
	6. African brush-tailed porcupine	
	7. Red river hog	
	8. Blue duiker	

190

Supplementary Table 4a: Palatability questionnaire used to collect data from 570 respondents in three categories (household, hunter, and wild meat vendor). The questionnaire we deployed via KoboToolbox (https://www.kobotoolbox.org/) installed in mobile tablets.

Question	Note
What is the name of the community?	The list of communities was determined before the survey.
What is the category of the respondent?	The categories were determined before the survey: hunter, household, and vendor.
What is the sex of the respondent?	
What is the year of birth of the respondent?	You can only interview people above 18 years. The form rejects any entry below 2006.
Which of these animals have you eaten meat from?	Here we handed the photos of the different species to participants to sort the animals whose meat they had eaten.
Rank the meat from these animals on a scale of 1-10, with 10 being most palatable and 1 being least palatable (give the meat from the animals wey you don chop numbers based on how much you like the taste, smell, and toughness of the meat.	The ranked meat refers to those they reported in the last step to have eaten. Here we provided cards numbered 1-10 to aid scoring.

Supplementary Table 4b: The list of meats included in the palatability questionnaire and the median palatability scores provided by respondents. Scores are sorted by meat type and then by taxonomic class for wild meat.

Species (Taxonomy)	Palatability score			
	Overall	Household	Hunter	Vendor
**Domestic meat**				
Cattle (*Bos taurus*)	8	8	7	7
Chicken (*Gallus gallus*)	9	9	9	10
Dog (*Canis faimiliaris*)	9	7	10	9
Duck (Anatidae)	5	6	5	5
Goat (*Capra hircus*)	8	8	8	7
Guinea pig (*Cavia porcellus*)	4	3.5	3.5	5
Pig (*Sus domestica*)	8	6	8	7
Rabbit (Leporidae)	6	5	8	6.5
Sheep (*Ovis aries*)	6	6	7	6
Turkey (*Meleagris* spp.)	7	6	7	7
**Fish**				
Anchovy (Engraulidae)	5.5	6	6	3
Carp (Cyprinidae)	8	9	9	7
Catfish (Siluriformes)	9	9	10	8
Sardine and pilchard (Alosidae)	5	4	5	7
Stock fish	7	7	8	6
Tilapia (*Oreochromis* spp.)	8	5	9	7
Mackerel (*Scomberomorus* spp.)	8	8	8	8
Invertebrate				
African palm weevil (*Rhynchophorus phoenicis*)	8	5	8	2
Aquatic snail (Melanopsidae)	7	7	6	7
Common periwinkle (Littorinidae)	7	7	7	7
Cricket (Grylloidea)	3	3	2.5	2
Freshwater crab (Potamidae)	5	5	5	6
Freshwater shrimp (Pleocyemata)	9	9	9	9
Giant African land snail (Achatinidae)	8.5	8	9	9
Grasshopper (Acrididea)	5	5	8	3

Lobster (Nephropidae)	8	7	9	8
Termite (Isoptera)	5	5	7	2
**<u>Wild meat</u>**				
<u>Bird</u>				
Black guineafowl (*Agelastes niger*)	6	5	7	6
Bush fowl (Numididae)	6	6	8	5
Eagle (Accipitridae)	5	2	6	6
Hawk and kite (Accipitridae)	4	3	4	5
Helmeted guineafowl (*Numida meleagris*)	7	7	8	7
Hornbill (Bucerotidae)	5	5	5	6
Owl (Strigiformes)	5	2	6	3
Parrot (Psittaciformes)	4	3	10	6
Pigeon (Columbiformes)	5	4.5	8	5
Small bird	4	3	4	1
Turaco (Musophagidae)	7	2.5	8	7
Vulture (Gypaetinae and Aegypiinae)	2	1.5	-	3
<u>Mammal</u>				
African brush-tailed porcupine (*Atherurus africanus*)	10	10	10	10
African buffalo (*Syncerus caffer*)	6	7	6	5
African civet (*Civettictis civetta*)	6.5	5	8	5
African elephant (*Loxondonta africana*)	8	7	8	8
African golden cat (*Caracal aurata*)	6	5	7	6
African palm civet (*Nandinia binotata*)	8	7	9	8
Bates' pygmy antelope (*Nesotragus batesi*)	7	7	8	7
Bats (Chiroptera)	5	5	5	2.5
Bay duiker (*Cephalophus dorsalis*)	7	6	7	7
Black-bellied pangolin (*Phataginus tetradactyla*)	10	10	10	10
Blue duiker (*Philantomba monticola*)	7	6	7	6.5
Bushbuck (*Tragelaphus scriptus*)	6	5.5	7	6

Calabar angwantibo (*Arctocebus calabarensis*)	5	5	4	5
Cameroon red-eared monkey (*Cercopithecus erythrotis*)	8	7	9	8
Chimpanzee (*Pan troglodytes*)	8	4	9	7
Crested genet (*Genetta cristata*)	7	6	7	6
Crested mona monkey (*Cercopithecus pogonias*)	8	7	8	7.5
Cross River gorilla (*Gorilla gorilla diehli*)	7	6	9	5
Cusimanse (*Crossarchus* spp.)	7	7	8	7
Drill (*Mandrillus leucophaeus*)	8	8	9	7
Galago (Galagidae)	5.5	5	5	6.5
Giant pangolin (*Smutsia gigantea*)	10	9.5	10	10
Giant pouched rat (*Cricetomys* spp.)	7	6	7	6
Greater cane rat (*Thryonomys swinderianus*)	9	9	10	8
Honey badger (*Mellivora capensis*)	4.5	5	7	4
Hyrax (Hyracoidea)	6	6	7.5	5
Mona monkey (*Cercopithecus mona*)	8	6	9	7
Mongoose (Herpestidae)	7	5	8	6
Ogilby's duiker (*Cephalophus ogilbyi*)	6	6	7	6
Otters (Lutrinae)	6	4	7.5	5.5
Patas monkey (*Erythrocebus patas*)	8	6	10	4
Milne-Edwards’s potto (*Perodicticus edwardsi*)	6	5	6	5
Preuss's red colobus (*Piliocolobus preussi*)	8	3	8	7
Preuss's monkey (*Allochrocebus preussi*)	7	3	9	6
Putty-nosed monkey (*Cercopithecus nictitans*)	8	6	9	7
Red river hog (Potamochoerus porcus)	10	9	10	8
Red-capped mangabey (*Cercocebus torquatus*)	8	5	9	8
Sclater’s monkey (*Cercopithecus sclateri*)	8	3	10	4

Shrew (Soricidae)	6	6	7	6
Sitatunga (*Tragelaphus spekii*)	7	5	7	7
Squirrel (Sciuridae)	6	5	7	6
Water chevrotain (*Hyemoschus aquaticus*)	7	6	8	7
White-bellied pangolin (*Phataginus tricuspis*)	10	10	10	10
Yellow-backed duiker (*Cephalophus silvicultor*)	5	3	6.5	6
<u>Reptile</u>				
African dwarf crocodile (*Osteolaemus tetraspis*)	7	6	8	6.5
Slender-snouted crocodile (*Mecistops cataphractus*)	8	7	9	6
Ball python (*Python regius*)	7	7	9	6
Central African rock python (*Python sebae*)	7	6	9	6
Chameleon (Chamaeleonidae)	5	5.5	10	2
Cobras (Elapidae)	6	6	7	5
Frog and toad (Anura)	6	5	7	6.5
Gaboon viper (*Bitis gabonica*)	7	6	8	6
Mamba (*Dendroaspis* spp.)	6	6	7	6
Nile crocodile (*Crocodylus niloticus*)	6	5	9.5	6.5
Nile monitor (*Varanus niloticus*)	8	7	8	8
Tortoise (Testudinidae)	7	7	8	5
Turtle (Testudines)	6	5	7	5

Supplementary Table 5: List of communities in the landscape used as the basis for our extrapolations. This list was compiled using GIS data from the Nigeria program of the Wildlife Conservation Society (WCS) and consultations with WCS staff for verification. WCS has over two decades of experience working in the Cross River Forest Landscape. Communities marked with an asterisk are located in Cameroon but lie on the edge of the park.

S/N	Community	Location
1	Abiang	Oban
2	Abiati	Oban
3	Abung	Oban
4	Acharum	Oban
5	Agbotai	Oban
6	Agoi Ekpo	Oban
7	Agoi Ibami	Oban
8	Akansoko	Oban
9	Akin	Oban
10	Akon	Oban
11	Aningeje	Oban
12	Ayukaba	Oban
13	Camp 3	Oban
14	Camp 4	Oban
15	Edondon	Oban
16	Efferaya	Oban
17	Ekang	Oban
18	Ekimaya	Oban
19	Ekong	Oban
20	Ekonganaku	Oban
21	Ekuri Eyeyeng	Oban
22	Esuk Aye	Oban
23	Etara	Oban
24	Ifumkpa	Oban
25	Iko Ekperem	Oban
26	Iko Esai	Oban
27	Ikpa	Oban
28	Iku	Oban
29	Isabang	Oban
30	Itaka	Oban
31	Iyamitet	Oban
32	Manko	Oban
33	Mbeban	Oban
34	Mberentung	Oban
35	Mfaminyen	Oban
36	Mfamosing	Oban
37	Mfum	Oban
38	Mkpot I	Oban
39	Mkpot Isong	Oban
40	Ndingane	Oban
41	Neghe	Oban
42	New Ekuri	Oban
43	New Ndebiji	Oban
44	New Nnetim	Oban
45	Nfamebine Akpama	Oban
46	Nkame	Oban
47	Nsan	Oban
48	Nsofang	Oban
49	Ntebachot	Oban
50	Nyaie	Oban
51	Oban	Oban
52	Oban Okoroba	Oban
53	Obarekkei	Oban
54	Obung	Oban
55	Ochon	Oban
56	Odonget	Oban
57	Ojok	Oban
58	Okarara	Oban
59	Okokuri	Oban
60	Okonoba	Oban
61	Okopedi	Oban
62	Okumurutet	Oban
63	Old Ekuri	Oban
64	Old Ekuri	Oban
65	Old Mfaminyen	Oban

66	Old Ndebiji	Oban	102	Danare	Okwangwo
67	Orem	Oban	103	Ebok	Okwangwo
68	Orem	Oban	104	Ebranta	Okwangwo
69	Osomba	Oban	105	Enyi	Okwangwo
70	Owai Ifumkpa	Oban	106	Ikwette	Okwangwo
71	Owom	Oban	107	Iso-Bendeghe	Okwangwo
72	Abo Bonabe	Okwangwo	108	Kajiku*	Okwangwo
73	Abo Mkpang	Okwangwo	109	Kakwe	Okwangwo
74	Abo Obisu	Okwangwo	110	Kanyang	Okwangwo
75	Abo Ogbagante	Okwangwo	111	Kanyang I	Okwangwo
76	Akwekia	Okwangwo	112	Katabang	Okwangwo
77	Amana	Okwangwo	113	Keji-Kwu	Okwangwo
78	Anapa	Okwangwo	114	Keyi	Okwangwo
79	Apagili	Okwangwo	115	Kigoh	Okwangwo
80	Ashishie	Okwangwo	116	Kundeve II	Okwangwo
81	Asuben	Okwangwo	117	Matere*	Okwangwo
82	Bagabo	Okwangwo	118	New Ekwete	Okwangwo
83	Bagga	Okwangwo	119	Njua Kaku	Okwangwo
84	Bago	Okwangwo	120	Nkanya	Okwangwo
85	Bajiki	Okwangwo	121	Obonyi 1*	Okwangwo
86	Bajomfue Bebo	Okwangwo	122	Obonyi 2*	Okwangwo
87	Bakie	Okwangwo	123	Obonyi 3*	Okwangwo
88	Bakuruku	Okwangwo	124	Obonyi*	Okwangwo
89	Bamba	Okwangwo	125	Ogbaitoko	Okwangwo
90	Bambariku	Okwangwo	126	Oguefor	Okwangwo
91	Bashu Kaku	Okwangwo	127	Ojoniya	Okwangwo
92	Bashu Okpame	Okwangwo	128	Okpazange	Okwangwo
93	Bateriko	Okwangwo	129	Okwa I	Okwangwo
94	Boggo	Okwangwo	130	Okwa II	Okwangwo
95	Boje	Okwangwo	131	Okwabang	Okwangwo
96	Bokalum	Okwangwo	132	Okwangwo	Okwangwo
97	Buabre	Okwangwo	133	Okwanu	Okwangwo
98	Buanchor	Okwangwo	134	Olum	Okwangwo
99	Bunu	Okwangwo	135	Onitsha Farm	Okwangwo
100	Butatong	Okwangwo	136	Oshonikpa	Okwangwo
101	Dadi*	Okwangwo	137	Otchakwe	Okwangwo

138	Takamanda*	Okwangwo
139	Ubong Alankwu	Okwangwo
140	Umbuli	Okwangwo
141	Utanga	Okwangwo
142	Wula I	Okwangwo
143	Wula II	Okwangwo
144	Yagwebe	Okwangwo

Supplementary Table 6: Median mass of whole, dried meat and scales of black-and white-bellied pangolins.

**Species**	**Part**	**Mass (in kg)**	**Sample size**
Black-bellied pangolin	Meat	1.820	2
Black-bellied pangolin	Scales	0.128	4
White-bellied pangolin	Meat	1.179	22
White-bellied pangolin	Scales	0.139	6

Extended Research Credits

Funding

Author salary: Bill & Melinda Gates Foundation (OPP1144; C.A.E.), University of Washington (S.K.W.), Center for International Forestry Research (L.C.), Dragon Capital Chair on Biodiversity Economics (B.B.), UK Research and Innovation's Global Challenges Research Fund (D.J.I.), CARE International UK (A.W.), Pangolin Protection Network (D.S.O., N.O.) and University of Cambridge (A.B.). **Data**: Protection Network through the Wildlife Conservation Society's Local Conservation Partners Fund, established with a grant from Arcadia—a charitable fund of Lisbet Rausing and Peter Baldwin. **Open access publishing**: University of Cambridge

Conceptualization

Idea formulation: C.A.E (lead) and A.B. Brainstorming: C.A.E. (lead), S.K.W., L.C., B.B., D.J.I., A.W., A.B. Concept development: C.A.E. (lead), A.B. Contextual guidance and coproduction: B.A.A., F.B., D.S.O., N.O., Nicholas Mbu Effa and Dan. O. Agbor. **Literature review**: C.A.E.

Data acquisition

Data collection: B.A.A., F.B., D.S.O., N.O., Nicholas Mbu Effa and Dan. O. Agbor. **Research operations coordination**: C.A.E. **Data contributor**: 809 anonymous volunteers.

Data processing and analysis

Cleaning and preprocessing: C.A.E. **Analysis: C.A.E. Tools (software)**: R statistical environment (v.4.2.242) within RStudio, lme4 (v.1.1.36), emmeans (v.1.11.0), performance (v.0.13.0), tidyverse (v.2.0.0), ggpubr (v.0.6.0), lubridate (v.1.9.4), ggsci (v.3.2.0), boot (v.1.3.31) packages, and QGIS (v.3.42).

Visualization

Illustration: Samudhi Silva and Anupama Dissanayake (Figure 3) and CAE. Map: C.A.E.

Manuscript

Original draft: C.A.E. **Internal review**: S.K.W., L.C., B.B., D.J.I., A.W., B. A.A., F.B., D.S.O., N.O., A.B. (lead). **External review**: Three anonymous reviewers. **Revision**: C.A.E.

Logistics

Research permit: C.A.E., Wildlife Conservation Society, Nigeria (facilitated approval). **Ethics**: C.A.E. and two anonymous reviewers. **Risk assessment**: C.A.E. and Sylviane Moss (reviewer).

Location

Location authorization: Nigeria National Park Service

Project management

Training (for data collection): C.A.E. **Funding management**: C.A.E. **Data management**: C.A.E. **Supervision**: A.B. **Quality control**: C.A.E, A.B.
