# Supplementary material for: Pangolin hunting in southeast Nigeria is motivated more by local meat consumption than international demand for scales
Source: Nat Ecol Evol. 2025 Jun 13;9(8):1349–58. doi: 10.1038/s41559-025-02734-3 (PMC12328206; doi:10.1038/s41559-025-02734-3)
Supplement: Supplementary file 2 — Reporting Summary [file 41559_2025_2734_MOESM2_ESM.pdf]

nature portfolio

Corresponding author(s): Charles A. Emogor

Last updated by author(s): 24/03/2025

Reporting Summary

Nature Portfolio wishes to improve the reproducibility of the work that we publish. This form provides structure for consistency and transparency in reporting. For further information on Nature Portfolio policies, see our Editorial Policies and the Editorial Policy Checklist.

Statistics

For all statistical analyses, confirm that the following items are present in the figure legend, table legend, main text, or Methods section.

n/a	Confirmed	
☐	☒	The exact sample size (*n*) for each experimental group/condition, given as a discrete number and unit of measurement
☐	☒	A statement on whether measurements were taken from distinct samples or whether the same sample was measured repeatedly
☐	☒	The statistical test(s) used AND whether they are one- or two-sided<br>*Only common tests should be described solely by name; describe more complex techniques in the Methods section.*
☐	☒	A description of all covariates tested
☐	☒	A description of any assumptions or corrections, such as tests of normality and adjustment for multiple comparisons
☐	☒	A full description of the statistical parameters including central tendency (e.g. means) or other basic estimates (e.g. regression coefficient) AND variation (e.g. standard deviation) or associated estimates of uncertainty (e.g. confidence intervals)
☐	☒	For null hypothesis testing, the test statistic (e.g. *F*, *t*, *r*) with confidence intervals, effect sizes, degrees of freedom and *P* value noted<br>*Give P values as exact values whenever suitable.*
☒	☐	For Bayesian analysis, information on the choice of priors and Markov chain Monte Carlo settings
☒	☐	For hierarchical and complex designs, identification of the appropriate level for tests and full reporting of outcomes
☒	☐	Estimates of effect sizes (e.g. Cohen's *d*, Pearson's *r*), indicating how they were calculated

Our web collection on statistics for biologists contains articles on many of the points above.

Software and code

Policy information about availability of computer code

Data collection	No proprietary software or custom code was used for data collection in this study. The code used for regression model fitting is available on GitHub at https://github.com/cemogor/pangolin-exploitation-in-southeast-nigeria.
Data analysis	All analyses were conducted using the R statistical environment (v.4.2.242) within the open-access integrated development environment, RStudio (https://posit.co/download/rstudio-desktop/). We used the lme4 package (v.1.1.36) to fit the models, emmeans (v.1.11.0) to perform post-hoc tests, and performance (v.0.13.0) to assess model fit. Data processing and visualization were carried out using tidyverse (v.2.0.0), ggpubr (v.0.6.0), lubridate (v.1.9.4) and ggsci (v.3.2.0). To estimate capture rates through bootstrapping, we used the boot package (v.1.3.31). The study map was created using QGIS (v.3.42).

For manuscripts utilizing custom algorithms or software that are central to the research but not yet described in published literature, software must be made available to editors and reviewers. We strongly encourage code deposition in a community repository (e.g. GitHub). See the Nature Portfolio guidelines for submitting code & software for further information.

Data

Policy information about availability of data

All manuscripts must include a data availability statement. This statement should provide the following information, where applicable:

- Accession codes, unique identifiers, or web links for publicly available datasets
- A description of any restrictions on data availability
- For clinical datasets or third party data, please ensure that the statement adheres to our policy

The data used in this study are available on Zenodo via this link: https://doi.org/10.5281/zenodo.15084096

Research involving human participants, their data, or biological material

Policy information about studies with human participants or human data. See also policy information about sex, gender (identity/presentation), and sexual orientation and race, ethnicity and racism.

Reporting on sex and gender	While our study relies on data from human participants, we did not include sex or gender as covariates in any of our models.
Reporting on race, ethnicity, or other socially relevant groupings	NA
Population characteristics	All research participants were either hunters, wild meat vendors, or other household members living in southeast Nigeria.
Recruitment	Our recruitment was systematic. To select 20 focal communities from 144 (14% of the total), we divided the two Cross River National Park divisions into four geographic quadrants (strata), including other protected areas. We randomly selected 12 communities (2-4 per stratum, except one strata where no community exists) and purposively sampled an additional eight communities where we were conducting research. Hunters were recruited at their homes, and vendors were recruited both at their homes and in wild meat markets.
Ethics oversight	We received ethics approval for this study from Cambridge University's Psychology Research Ethics Committee (application number: PRE.2023.097). Study participants provided written, free, and informed consent before we commenced the survey and all data were anonymised.

Note that full information on the approval of the study protocol must also be provided in the manuscript.

Field-specific reporting

Please select the one below that is the best fit for your research. If you are not sure, read the appropriate sections before making your selection.

☐ Life sciences ☒ Behavioural & social sciences ☐ Ecological, evolutionary & environmental sciences

For a reference copy of the document with all sections, see nature.com/documents/nr-reporting-summary-flat.pdf

Behavioural & social sciences study design

All studies must disclose on these points even when the disclosure is negative.

Study description	We used a mixture of self-reported qualitative and quantitative data for this study, some of which we verified using an independent dataset from the same region.
Research sample	The data used in this study were collected in 2023. The core data came from 809 local hunters and wild meat vendors in southeast Nigeria, with supplementary data on meat palatability from 570 hunters, vendors, and households in the same landscape. Cross River forest landscape, in southeast Nigeria, is one of the largest remaining forest blocks in the Guinean Forest biodiversity hotspot. The landscape is a hotspot for pangolin scale exports, as well as a stronghold for African pangolins in Africa. Nigeria is a major hub in the global, illegal pangolin trade.
Sampling strategy	Our recruitment was systematic. To select 20 focal communities from 144 (14% of the total), we divided the two Cross River National Park divisions into four geographic quadrants (strata), including other protected areas. We randomly selected 12 communities (2-4 per stratum, except one strata where no community exists) and purposively sampled an additional eight communities where we were conducting research - some of the additional communities were randomly selected. Hunters were recruited at their homes, and vendors were recruited both at their homes and in wild meat markets.<br><br>After informing community leaders of our study and requesting their permission to conduct the surveys, we counted all households in each community, defining a household as a group of people living under the same roof and sharing the same meals. We aimed to sample all hunters and vendors in each community, so during the count we asked if a household member was a hunter or vendor. We then returned to households with our respondents of interest to go through the questionnaire with them individually (note that some hunters and vendors declined to take part, and others were absent). We then recruited additional vendors through market

	visits.<br><br>We followed a similar approach in recruiting respondents for the palatability survey.
Data collection	Data were collected using a standardized survey on Kobotoolbox (https://www.kobotoolbox.org/) via Samsung tablets. Interviews were held with individuals not in groups.
Timing	The core data were collected in October to November 2023, with palatability data gathered in August-September 2022.
Data exclusions	In our linear model of price of animal part over time, of the 809 respondents, we used data from 431 respondents, as we dropped those who did not provide prices for either meat or scales for all four periods: i) January 2010-December 2015; ii) January 2016-February 2020; iii) April-September 2020 (COVID-19 lockdown in Nigeria); and iv) October 2020-September 2023. Note also that we already discarded 80 records where scale prices were incorrectly collected on a per kilogram instead of per animal basis.
Non-participation	No participant dropped from the study. However, 19 people declined to take part.
Randomization	We randomly selected focal communities after overlaying four strata in each of the two divisions of the Cross River National Park, the largest protected area in the landscape. Further, we applied systematic approach in gathering data from participants (except the vendors recruited from wild meat markets).

Reporting for specific materials, systems and methods

We require information from authors about some types of materials, experimental systems and methods used in many studies. Here, indicate whether each material, system or method listed is relevant to your study. If you are not sure if a list item applies to your research, read the appropriate section before selecting a response.

Materials & experimental systems

n/a	Involved in the study
☒	☐ Antibodies
☒	☐ Eukaryotic cell lines
☒	☐ Palaeontology and archaeology
☒	☐ Animals and other organisms
☒	☐ Clinical data
☒	☐ Dual use research of concern
☒	☐ Plants

Methods

n/a	Involved in the study
☒	☐ ChIP-seq
☒	☐ Flow cytometry
☒	☐ MRI-based neuroimaging

Plants

Seed stocks	NA
Novel plant genotypes	NA
Authentication	NA
